# On the Design of a Sign Language Corpus of Medical Terms for Automatic Translation Systems: Mixed Methods Approach

**DOI:** 10.2196/72789

**Published:** 2026-04-29

**Authors:** Milena Soriano Marcolino, Luiza Marinho Motta Santa Rosa, Elidea Lucia Almeida Bernardino, Mario Fernando Montenegro Campos

**Affiliations:** 1 Medical School and Telehealth Center, Universidade Federal de Minas Gerais, 190 Professor Alfredo Balena Ave, Belo Horizonte, 30130-100, Brazil, 55 3133079201; 2 Faculdade de Ciências Médicas de Minas Gerais, Belo Horizonte, Brazil; 3 Faculty of Arts & Sciences, Universidade Federal de Minas Gerais, Belo Horizonte, Brazil; 4 Department of Computer Science, Universidade Federal de Minas Gerais, Belo Horizonte, Brazil

**Keywords:** deafness, sign language, assistive technology, emergency health services, Delphi technique

## Abstract

**Background:**

Hearing loss is a global health issue affecting millions and creating significant communication barriers, particularly in accessing health care services. These barriers can lead to complications and iatrogenic events, emphasizing the need for assistive technologies that enhance communication efficiency.

**Objective:**

This study aimed to develop a corpus of medical terms for the “Captar-Libras” project, designed to improve communication between health care professionals and deaf patients through a bidirectional sign language system.

**Methods:**

This study used the Delphi method to obtain consensus on key terms for a sign language translation system in health care emergency consultations. Initially, a questionnaire with common emergency questions was developed and distributed to health care professionals. The collected data were analyzed by a team of experts and adapted to Brazilian Sign Language (Língua Brasileira de Sinais [Libras]). Simulated clinical scenarios were then created to validate the system and ensure the accuracy of the vocabulary in the medical context.

**Results:**

Among the 16 participants, most were physicians (n=14, 87.5%) with experience in emergency care, and half had previously treated patients with hearing loss in emergency settings. The questions evaluated received high average importance scores, particularly those related to initial symptoms and pain intensity. Some suggestions for adjustments were made, with two wording modifications significantly improving clarity regarding smoking and alcohol use. Additional suggestions to enhance the medical interview were also proposed. This study aimed to identify essential questions for emergency consultations with deaf patients, focusing on developing a corpus for Libras recognition system. The findings emphasize the importance of effective communication and highlight the challenges of translating medical terms into Libras. To address these complexities, a multidisciplinary team used the Delphi method to ensure linguistic and cultural accuracy. Additionally, the study reinforces the need for clear, structured medical queries to improve accessibility in emergency care. As a next step, system validation through simulated scenarios will be conducted. Despite certain limitations, this research lays a solid foundation for advancing sign language recognition in medical settings.

**Conclusions:**

This study represents a significant methodological step toward improving communication between health care professionals and deaf individuals in emergency medical settings. Rather than proposing a universal solution, the study presents a structured and participatory approach for developing a corpus of medical terms in Libras, with interdisciplinary validation. The process included the involvement of deaf sign language experts during the translation and linguistic adaptation phase, ensuring that the corpus reflects authentic usage and articulation in Libras.

## Introduction

Hearing loss is a significant global health issue, affecting millions worldwide. According to the World Health Organization (WHO), more than 430 million people globally experience some degree of disabling hearing loss, a number projected to rise to 700 million by 2050 [1]. Approximately 10 million people in Brazil are deaf, 2.6 million of whom have severe functional difficulties [2]. For these individuals, sign language is not just a communication tool but a vital aspect of their cultural identity, enabling interactions based on gestures and facial expressions.

Despite the critical role of sign language in the lives of deaf individuals, significant communication barriers persist, particularly in accessing health care. These barriers are a reflection of the broader challenges faced by people with disabilities, who often encounter services that are not equipped to meet their specific needs [3]. In the context of health care, these challenges can have profound implications. The lack of effective communication between health care providers and deaf patients can lead to misdiagnosis, inappropriate treatments, and overall poorer health outcomes [4].

The situation is even more challenging in emergency settings, where the inability to establish timely and effective communication can directly impact the health and safety of deaf individuals. Delays in diagnosis, treatment, and even basic information exchange can result in worse prognosis, higher incidence of complications, and iatrogenic events [4,5]. This underscores the urgent need for improved accessibility and preparedness within health care systems to address the needs of deaf individuals.

To address some of these challenges, the project “Captar-Libras: Video Communication System for the deaf applied to medical pre-care” from the Universidade Federal de Minas Gerais (UFMG) aims to overcome this obstacle by developing a prototype for the recognition and synthesis system for Brazilian Sign Language (Língua brasileira de sinais [Libras]), with the recognition of signs and generation of gestures and expressions with a photo-realistic avatar.

For successful implementation and future applicability, it was first necessary to select the most relevant terms and questions most commonly used in medical consultations. Freitas [6] defines “corpus” as a collection of text documents compiled according to a specific objective that can be processed by machines. Thus, Corpus Linguistics plays an important role in enabling the development of new assistive technologies, especially in the construction of databases for the study of languages [7].

However, in a systematic review conducted by our group, we found that no studies have been published regarding the development of such a corpus of questions and possible answers to be used in translation systems [8]. Therefore, this study aims to construct a linguistic corpus specifically tailored for emergency medical care, serving as the foundational resource for the development of real-time sign language translation technologies to improve health care accessibility for the deaf community. We hypothesize that the development of a medical corpus based on commonly used terms and questions in emergency consultations will provide the necessary linguistic foundation for effective and accurate sign language translation systems.

## Methods

### Study Design

This study used a qualitative, exploratory, and consensus-based Delphi method within a mixed methods framework. It constitutes a subanalysis of the “Captar-Libras Project”—a multidisciplinary initiative focused on developing a bidirectional sign language recognition and translation system for primary medical consultations. Recognizing the paucity of literature on sign language translation systems in health care, the Delphi method was selected for term selection. Initially conceptualized in the 1950s, this research strategy effectively elicits expert consensus on subjects where scientific evidence is insufficient or divergent [9].

#### Step 1: Initial Question Formulation

The process began by identifying the most frequent questions encountered in emergency care settings. This was based on the extensive experience of the lead researcher (MSM), who had more than 12 years of experience in emergency care. Drawing from this expertise, a preliminary set of questions was developed to reflect the most common and critical inquiries health care professionals need to communicate with patients during emergencies.

#### Step 2: Questionnaire Development

Using the initial set of questions devised in Step 1 as a foundation, a detailed questionnaire was created. The questionnaire included items developed through a Delphi approach to identify and reach consensus on the most relevant medical terms and questions for emergency care interactions with deaf patients, using Likert scale items. It also included open-ended questions to gather qualitative feedback. This mixed methods approach ensured that the final selection of terms and questions would meet the practical needs of a consultation at the emergency care service. Then, the questions were organized and clustered to provide the respondents with a natural flow and facilitate the data collection process, which was carefully planned to minimize the time and burden of answering the questions and to optimize communication between our team and the participants.

#### Step 3: Questionnaire Content

The questionnaire consisted of 6 questions regarding the professional profile, 21 Likert scale questions, and 2 open-ended questions. Besides the questions defined and established in Step 1, there were also questions designed to evaluate the participants’ experience with emergency care and interacting with deaf patients (Multimedia Appendix 1). One of the questions assessed the professionals’ workplace, with options: “hospital”—large health establishments with an organized flow of care, “emergency care unit”—medium-complexity services with open access whose objective is to provide initial assistance in urgencies or emergencies, and “pre-hospital care”—which involves the first care and transportation to hospitals.

#### Step 4: Data Collection

The questionnaire was created in Google Forms (Google LLC) for easy access and availability, and the link was sent via WhatsApp (Meta Platforms, Inc), a widely popular instant messaging app in Brazil. Before answering, the participants received a brief overview of the “Captar-Libras Project” and an informed consent form.

##### Expert Selection

Experts were recruited among physicians and nurses currently working or with prior professional experience in emergency care services. This criterion was adopted because familiarity with emergency consultations is essential for identifying the most relevant questions and medical terms to be included in the corpus.

### Step 5: Data Analysis

After the data collection period ended, responses were analyzed to identify consensus on the most critical terms and the need to include additional questions for the sign language recognition corpus. Consensus was defined a priori as at least 80% of participants rating an item as 4 or 5 on the 5-point Likert scale. Nevertheless, all qualitative suggestions provided in the open-ended questions were also considered. The team of linguists and Libras specialists evaluated these contributions to determine their applicability to sign language translation, ensuring linguistic and cultural adequacy. Once the final corpus was consolidated in Brazilian Portuguese, adjustments were carried out for translation and adaptation into Libras.

Quantitative data from the Likert scale questions were analyzed using descriptive statistics. Specifically, the proportion of participants selecting each response option was calculated for each question. No inferential statistical tests were conducted, as the primary aim was to identify consensus and assess the relative importance of terms for inclusion in the sign language corpus.

While the Delphi process established the selection and prioritization of questions, the response options were constructed based on the expertise of the lead author (MSM) to reflect realistic scenarios and ensure practical applicability. This approach allowed the incorporation of clinically accurate and contextually appropriate responses, which were subsequently adapted into Libras during the translation and validation process.

### Step 6: Adaptation Process for Sign Language

The sign language team carefully analyzed the corpus in Portuguese for translation and cultural and linguistic adaptation. A direct word-for-word translation was not feasible due to the structural and semantic specificities of sign languages. Therefore, it was essential to make adaptations and adjustments in the corpus to ensure that the corpus reflected how deaf users articulated specific medical terms. The initial adaptation team included 2 deaf individuals, a sign language linguist (university professor), and a graduate student with health-related experience, and 2 hearing professionals (a sign language linguist fluent in Libras and a sign language interpreter). The team worked collaboratively to identify terminological discrepancies between spoken Portuguese and Libras, considering both linguistic and contextual aspects.

The adaptation process followed an iterative cycle:

1. Initial translation and restructuring: the team identified questions that were too complex for direct translation and restructured them into shorter, sequential items (eg, the cluster “If the complaint is pain: Where is the pain? What is the pain like? Does it feel like a tightness or weight, or burning, or stabbing, or a shock?” was expanded into multiple questions regarding location, type, and sensation). Some concepts required additional explanation or modification; for example, (1) “Pain in the middle of the belly” was clarified by medical staff for accurate articulation. (2) The term “trauma” lacks a direct sign, so translations were elaborated to convey “physical trauma” or “psychological trauma” depending on context. (3) “Penetrating trauma” was translated into a descriptive sign sequence (“material group cut also long thin object stick”) to ensure comprehension. (4) Aggression was translated using the most comprehensive and contextually appropriate signs, reflecting various scenarios of physical aggression. Decisions were guided by the principle that the translated signs should reflect the intended meaning, be understandable by deaf informants, and align with authentic Libras usage. This approach is consistent with previous work highlighting that effective communication in health care requires corpora that are functionally and contextually realistic, thereby improving the accuracy and usability of sign language recognition systems [10].
2. Peer validation: each item was translated to Libras by a deaf undergraduate student and recorded as individual videos. To validate comprehension, the videos were presented to a small group of deaf graduates and to a deaf university professor who had not seen the questionnaire in Portuguese. Their feedback focused on clarity, naturalness, and cultural adequacy.
3. Revisions: terms or expressions flagged as unclear were reformulated. For example, grammatical suggestions that had no functional equivalent in Libras were discarded, while ambiguous medical terms were replaced with more visually descriptive alternatives.
4. Final validation: this cycle of translation, feedback, and revision was repeated until mutual comprehension was reached across both deaf linguists and health professionals.

During the project, team composition evolved (the deaf professor left for personal reasons and was replaced by another deaf linguistics professor, while new members, including a physiotherapist and 2 undergraduate students, joined), but the iterative approach remained constant. Throughout the adaptation process, all feedback from deaf linguists, health professionals, and volunteer reviewers was carefully considered. Suggestions that enhanced clarity, comprehension, or cultural accuracy were incorporated directly into the corpus, while minor issues irrelevant to sign language translation (eg, Portuguese punctuation) were documented but not applied. This iterative approach ensured that each input led to specific refinements, resulting in a final corpus that accurately reflected the intended medical terminology in Libras and was understandable by the target population. Overall, the adaptation phase spanned approximately 3 months, encompassing reevaluation, translation, and video recording, culminating in the production of a definitive video corpus. The final version served as a “teleprompter” guide for deaf volunteers, who reproduced the validated signs in a controlled setup composed of multiple cameras, chromakey background, and illumination. The data collection started in January 2024 and was finalized in May 2025.

### Step 7: Development of Hypothetical Medical Care Situations

Hypothetical clinical scenarios were developed by the aforementioned researcher (MSM), who has clinical expertise in emergency care, based on common situations encountered in emergency settings. Each scenario was designed to simulate realistic interactions between health care professionals and deaf patients, including typical questions about symptoms, pain, and treatment history. This step was fundamental to ensure adequate understanding by the systems team, enabling the translation into sign language process to be carried out without losing critical information or meaning.

The evaluation of the sign language recognition system, which is being developed, will focus on three aspects:

1. Comprehension by the target users: deaf volunteers will assess whether the terms and questions are clearly understood and reflect realistic emergency situations.
2. Accuracy of translation: the system’s ability to correctly interpret and reproduce the intended medical terms in Libras will be checked against the original Portuguese questionnaire.
3. User experience and system performance: participants will provide feedback on ease of use, clarity of interaction, and any technical issues such as system crashes or delays, which will inform iterative improvements to the interface and functionality.

Feedback from participants will be systematically recorded, and necessary adjustments will be made iteratively to ensure the clarity, accuracy, and usability of the system in emergency care contexts.

### Ethical Considerations

This study was approved by the UFMG Ethics Review Board, under protocol number CAAE 71623323.8.0000.5149, in accordance with national and international research ethics guidelines. All participants, including health care professionals and sign language experts, provided informed consent prior to participation in the Delphi process, translation, and validation activities. In addition to obtaining informed consent, all procedures were designed to respect cultural and linguistic nuances within the deaf community. The participants’ autonomy, comfort, and comprehension were prioritized during data collection. Data collected through questionnaires were anonymized to ensure confidentiality. Video recordings were securely stored, accessible only to the research team, and were used exclusively for the purposes of corpus development. Participants and the team members who appeared in video recordings of sign language production signed specific consent forms authorizing the use of their images for research purposes and for inclusion as multimedia material accompanying this publication. No other personally identifiable information is disclosed in this paper or in the supplementary material. Participants who were deaf and took part in the video recordings received financial compensation to cover transportation costs to the UFMG, where the recordings took place.

## Results

### Profession and Field of Activity

Among the 16 participants who answered the questionnaire, 12/16 (75%) were currently working in emergency and urgent care services, which aligns with the focus of this study. Among these 12 participants, 11/12 (91.67%) were physicians and 1/12 (8.33%) was a nurse. Considering all participants (N=16), 3/16 (18.75%) participants had worked in the area for less than 5 years, 4/16 (25%) for 5 to 10 years, and 5/16 (31.25%) for more than 10 years. Regarding the workplace, 7/16 (43.75%) participants reported working in hospitals, 2/16 (12.5%) in emergency care units, 1/16 (6.25%) in prehospital care, and 2/16 (12.5%) worked simultaneously in hospitals and emergency care units.

### Experience in Treating a Hearing-Impaired Patient in an Emergency

Among the 12 participants working in emergency and urgent care services, 8 (66.66%) had already treated hearing-impaired patients in emergencies. Regarding the frequency of these occurrences, 2/12 (16.67%) professionals had treated 2 patients in this scenario, 4/12 (33.33%) had treated between 2 and 5, and 2/12 (16.67%) had treated more than 5.

### Level of Relevance of the Proposed Questions

**Figure 1.**
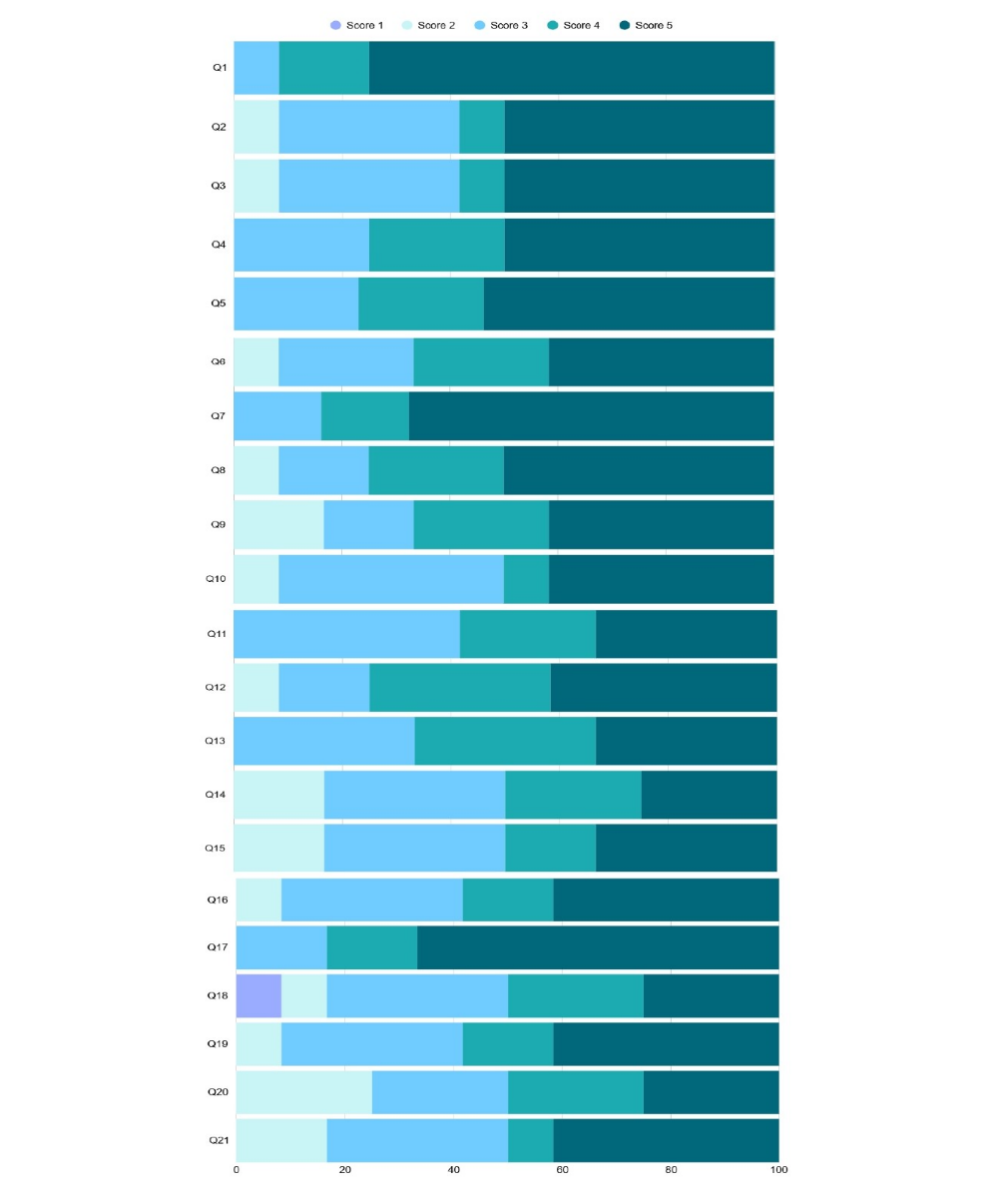
Graphical representation of the importance score assigned to each question by health professionals with emergency care experience.

Figure 1 and Multimedia Appendix 2 summarize the general results. Most questions were considered important, with medians predominantly between 4 and 5. Notably, questions addressing the primary complaint (eg, “What are you feeling that made you seek the hospital?”) and the patient’s medical history (eg, “Are you allergic to any medication?") were consistently rated as highly relevant.

A Likert scale ranging from 1 to 5 was used, with the following correspondence: 1=not important at all; 2=of little importance; 3=of average importance; 4=very important; 5=absolutely essential.

### Opinion on the Wording of the Questions and Contact Availability

Six participants suggested 10 recommendations for improvement of the already proposed questions (Textbox 1). Most suggestions involved minor grammatical adjustments, which were deemed nonrelevant for sign language translation by the team of linguistics specialists. Questions regarding smoking and alcohol consumption were revised to better capture the patients’ habits, reflecting these contributions. The other 6 participants indicated no need for changes.

Textbox 1. **Questions included in the final version for the corpus development.**

1. If the complaint is pain: was it 1 episode of pain, or more episodes? If more episodes, when was the first episode? How did the pain evolve? Did it increase in intensity? Did it increase in duration?
2. Are you feeling nauseous or vomiting? Do you have diarrhea? If so, what is it like? Is there blood, mucus, or pus? Do you have stomach pain?
3. Have you had any surgery? If so, which one?

### General Suggestions and Proposals for New Questions

Five participants provided other general suggestions or proposed new questions:

1. Incorporating the Manchester Protocol for prioritizing patient care
2. Organizing the assessment in a flowchart format, based on the main complaint and the Manchester protocol
3. Inserting a question regarding the duration of the pain and its evolution (whether it has improved or worsened over time)
4. Inserting a question regarding other known allergies and surgical history
5. Inserting questions regarding gastrointestinal complaints (“Are you feeling nauseous or vomiting? Do you have diarrhea? Do you have stomach pain?”)

Although the inclusion of the Manchester Protocol and organizing in a flowchart assessment would be enriching, these suggestions exceeded the scope of this study. In the case of the Manchester Protocol, information about waiting time depending on the severity of the patient’s pain is relevant for health care professionals. For patients, this is not a piece of information that needs to be translated into sign language. Regarding the other suggestions to insert new questions, the changes were made (Table 1).

**Table 1.** Initial questions with their respective modifications and sign language videos.

Proposed question	Version after Delphi method	Final version after team correction
1. What are you feeling that made you seek the hospital?	No suggestions made	What are you feeling that made you seek the hospital?
2. When did it start?	No suggestions made	When did it start?
3. How did it start? What were you doing when it started?	How did it start? What were you doing the moment it started?	How did it start? What were you doing when it started?
4. How long has it lasted?	No suggestions made	How long has it lasted?
5. Do you feel anything else, besides this symptom?	No suggestions made	Do you feel anything else, besides this symptom?
6. Is there any factor that makes it worse, or better?	Is there any factor that makes it worse or better?	Is there any factor that makes it worse or better?
7. If the complaint is chest pain or shortness of breath: does it get worse when you make a physical effort? Climbing a hill, walking on a flat surface, or taking a shower? Or does it appear when you are at rest, without any effort? Has it improved with any medication?	No suggestions made	If the complaint is chest pain or shortness of breath: does it get worse when you make a physical effort? Climbing a hill, walking on a flat surface, or taking a shower? Or does it appear when you are at rest, without any effort? Has it improved with any medication?
8. If the complaint is pain: Where is the pain? What is the pain like? Does it feel like a tightness or weight, or burning, or stabbing, or a shock?	No suggestions made	If the complaint is pain: Where is the pain? What is the pain like? Does it feel like a tightness or weight, or burning, or stabbing, or a shock?
9. On a scale of 1 to 10, with 1 being very weak pain and 10 being unbearable pain, what is the intensity of the pain?	No suggestions made	On a scale of 1 to 10, with 1 being very weak pain and 10 being unbearable pain, what is the intensity of the pain?
10. Does it stay in one fixed place, or does it spread to another location?	Does it stay in one fixed place or does it spread to another location?	Does it stay in one fixed place, or does it spread to another location?
11. Does it get worse when you press? Does it get worse when you change your body position?	Does it get worse when you press or compress it? Does it get worse when you change your body position (or move your body)?	Does it get worse when you press? Does it get worse when you change your body position?
12. If you complain of fever or chills, do you have sneezing or nasal discharge?	No suggestions made	If you complain of fever or chills, do you have sneezing or nasal discharge?
13. Do you have a cough? If so, what is the cough like, productive or dry? What color is the phlegm?	Do you have a cough? If so, what is the cough like, productive or dry? What is the phlegm’s color?	Do you have a cough? If so, what is the cough like, productive or dry? What color is the phlegm?
14. If you don’t sneeze or cough, do you feel a burning sensation when urinating?	If you don’t sneeze or cough, does it burn when urinating?	If you don’t sneeze or cough, do you feel a burning sensation when urinating?
15. If you complained of fever, chills, cough, shortness of breath, or if you said you had sneezing or nasal discharge: have you been vaccinated against COVID-19? If so, how many doses? Have you been vaccinated against the flu?	If you complained of fever, chills, cough, or shortness of breath, or if you said you had sneezing or nasal discharge: were you vaccinated against COVID-19? If so, how many doses? Have you been vaccinated against the flu?	If you complained of fever, chills, cough, shortness of breath, or if you said you had sneezing or nasal discharge: have you been vaccinated against COVID-19? If so, how many doses? Have you been vaccinated against the flu? Did you bring your vaccination card?
16. Are you being treated for any disease? If so, which one?	No suggestions made	Are you being treated for any disease? If so, which one?
17. Are you allergic to any medication? If so, which one?	No suggestions made	Do you have any allergies? Are you allergic to any medication? If so, which one?
18. Did you bring a medical report?	Did you bring a medical document?	Did you bring a medical report?
19. Did you bring a prescription? If not, are you taking any medication?	No suggestions made	Did you bring a prescription? If not, are you taking any medication?
20. Do you smoke?	Do you smoke or have you ever smoked? If so, for how long and how many cigarettes per day?	Do you smoke? If so, for how long and how many cigarettes per day?
21. Do you drink alcohol? If so, how much?	Do you drink alcohol? If so, which drinks and how much?	Do you drink alcohol? If so, which drinks and how much?

Besides the listed changes, some newly proposed questions were included and are shown in Textbox 1.

The final version of the questions and their respective videos in sign language (Libras) is shown in Multimedia Appendix 3.

### Hypothetical Medical Care Situations

Fourteen hypothetical scenarios were developed as a corpus enhancement tool in order to test its applicability and comprehensiveness (Multimedia Appendix 4). The medical conditions were selected according to their prevalence in emergency services, in a way that the main diagnosis involved could be broadly represented. Therefore, the scenarios included common infections, inflammatory conditions such as acute abdomen, migraine, respiratory decompensation, cardiovascular, and thromboembolic events. In each of them, a clinical description of the patient’s probable complaints and expected physical examination data was made. This stage was essential for the corpus’s validation process.

## Discussion

### Main Findings

This study demonstrated the feasibility and methodological value of using a Delphi technique to develop a clinically relevant corpus of medical terms. In this analysis, the process focused on emergency care and involved health care professionals to identify and refine expressions considered essential for patient-provider communication. The subsequent linguistic adaptation phase engaged deaf and hearing sign language experts to ensure that the selected content was accurate, natural, and culturally appropriate in Libras. The final corpus includes questions and expressions organized to reflect the logical sequence of emergency medical anamnesis, thus bridging clinical communication needs with the linguistic and cultural norms of the deaf community. These findings highlight the potential of interdisciplinary, consensus-based approaches to improve accessibility and guide the development of sign language resources and technologies in health care. They also underscore the complexities involved in developing a bidirectional translation system that takes into account the linguistic and cultural specificities of the deaf community.

The need for this study arose from a larger project, whose objective is to develop a prototype for recognizing Brazilian sign language in the context of medical settings [11]. This area is still largely underexplored, with a growing demand for development based on emerging technological innovations and the use of novel artificial intelligence models.

### Comparison With Prior Work

While the development of translation systems for sign languages has been explored in prior research, and some studies have described the creation of sign language corpora for health care–related applications, none have used a structured consensus method such as the Delphi technique. For example, Camgöz et al [12] developed a Turkish Sign Language Recognition Corpus that included content from health, finance, and daily life domains. Although the authors consulted linguists, members of the deaf community, and domain specialists, they did not report how the final corpus content was selected. Similarly, Wille et al [13] described the development of a Flemish Sign Language corpus based on descriptive grammatical research, including elicited and spontaneous narratives, conversations, and on-topic interviews. However, these corpora were primarily linguistically oriented, with no focus on health care, and did not involve systematic validation by health care professionals. Roelofsen et al [14] reported collecting a set of phrases commonly used during the diagnosis and treatment of COVID-19, based on consultations with health care professionals at the Amsterdam University Medical Center as well as on direct experience (one of the authors is a medical doctor). They also referenced phrases from the SignTranslate system. However, the authors were not transparent about how the most important sentences were selected or ranked for inclusion in the corpus [14].

In a previous systematic review of sign language translation systems, the corpus development process was not even mentioned [15]. Our recent systematic review identified 23 studies involving sign language recognition systems developed and tested to be applied in health care settings [8]. These studies often emphasize technical aspects, such as gesture recognition, but provide little detail on how the linguistic content, particularly in specialized domains such as health care, is selected or validated. The vast majority were in the development or testing stage and used corpora based on isolated terms, often defined by researchers or adapted from dictionaries, with minimal contextual adaptation. Very few studies involved health care professionals or sign language experts in corpus development, and none used structured consensus methods such as the Delphi technique. Furthermore, most systems lacked integration of key communicative features such as facial expressions and bidirectionality, both essential for health care communication. To our knowledge, no prior study has detailed a participatory, interdisciplinary approach to constructing a medically oriented sign language corpus using both clinical and linguistic validation. Our study addresses this gap, contributing a novel methodology tailored to emergency health care settings in Libras.

The lack of literature on this specific process led us to create approaches that seemed to be most adequate. After much investigation and planning, the Delphi method was selected [9], since it could assist in directing and supporting the process of choosing terms, thus ensuring its reproducibility. With the collection and analysis of the responses, the process of adaptation to sign language was carried out in collaboration with the linguistics team.

### Relevance of Proposed Questions

The high relevance ratings for most of the proposed questions (with medians predominantly between 4 and 5 on the Likert scale) reflect the importance of accurate, detailed, and efficient communication in emergency care. Specifically, questions related to the patient’s primary complaint (eg, “What are you feeling that made you seek the hospital?”) and medical history (eg, “Are you allergic to any medication?”) were consistently rated as critical. This aligns with the standard practices in emergency medical care, where understanding the nature and timeline of symptoms is essential for accurate diagnosis and appropriate treatment [16].

The consistency in the importance assigned to these questions indicates that health care professionals value information that directly impacts clinical decision-making. This also reinforces the necessity for such questions to be clearly and accurately translatable into sign language, ensuring that deaf patients can provide this vital information without delay or confusion.

### Challenges in Sign Language Translation

One of the main challenges identified in this study was the translation of medical terms and questions into Libras. As sign languages are linguistically distinct from spoken languages, direct translation of phrases often leads to ambiguities or misinterpretations [17]. The need for careful adaptation, particularly for complex medical queries (eg, questions regarding the nature of pain or its progression), was emphasized by the research team. In many cases, questions had to be broken down into simpler, shorter queries, a process that aligns with previous findings in the literature regarding the importance of adapting questions for clarity in sign language. This process, while time-consuming, is crucial to ensure that the information exchanged during an emergency consultation is accurate and comprehensive.

When translating into Libras, 2 questions were more challenging, for different reasons. The first of them, question 9, “On a scale of 1 to 10, with 1 being very weak pain and 10 being unbearable pain, what is the intensity of the pain?” It has a more complex grammatical structure, and it was not possible to break it into 2 or more distinct questions, as we did with question 8, for example. As a translation strategy, we transformed the question into a comment topic. In other words, we start the sentence with the topic “pain,” followed by the question-comment “on a scale from 1 to 10, with intensity 1 being a weak pain, intensity 5 being a medium pain, and 10 being a very strong pain, what is the intensity of the pain?” The addition of “average pain” gave the question a better idea of the intended gradation.

In the second question 10, “Does it stay in one fixed place, or does it spread to another location?,” the challenge was to define a pain location without interfering with the original question. This is because “[to] stay in one fixed place” makes no sense in Libras if it is not defined in the signer’s body what that place is. The translation choice of the deaf woman who recorded the question was “pain, for example [points to a place on her own body], stays in the same place or spreads [performs the sign [to spread] starting from the chosen location on her body for the pain and spreading to adjacent places].”

These translation strategies were discussed with the translation team, tested, and evaluated until we reached a consensus on what would be the best way to translate them without interfering with the desired meaning, but taking into account how the deaf person would clearly understand.

Additionally, it became evident that translation is not merely a matter of substituting words; it involves a deep understanding of both the linguistic structure of sign language and the cultural context in which it is used [17].

The involvement of a multidisciplinary team, including deaf linguists, health professionals, and sign language interpreters, was essential to ensure the validity and appropriateness of the translations. The medical professionals on the team were essential in the initial process of suggesting sentences, which, after evaluation by the group of health professional experts, were submitted to the linguistics team for careful evaluation. This framework ensured that all necessary adjustments were made to guarantee that the final product was scientifically and grammatically correct, both in the written and signed versions.

Surely much can be learned from the advances in natural language processing; however, there are many new dimensions yet to be explored when it comes to sign language understanding and synthesis. If, on the one hand, spoken languages embed significant information such as rhythm, tone, and pauses, on the other hand, it does not require delving into finer details such as facial expressions, as is the case with sign languages. When describing the creation of a text-to-sign translation method, Bertin-Lemée et al [18] mention the need to preserve the linguistic structures of sign language during the translation process and point it out as an inherent challenge to the process.

This stage involved making the necessary adjustments to the sentences to ensure their correct meaning in both written and signed forms. All suggestions were carefully analyzed, with several related to the positioning of commas and grammatical structure, without significantly interfering with the message conveyed or the intent of the questions. It is important to note that those unfamiliar with sign language may not realize that punctuation, such as commas, does not hold the same relevance in this context. The structure of sign language differs significantly from spoken languages, and such differences are often overlooked by society at large [17]. Only 2 suggestions were deemed significant, leading to adjustments in questions 20 and 21. This highlights the importance of having knowledgeable researchers with extensive technical expertise to ensure a proper critical analysis and adaptation of the content.

### Implications for Practice

This project has significant implications for improving health care accessibility for deaf individuals, especially in emergency care settings. By developing a system that enables real-time communication in Libras, the “Captar-Libras” Project aims to reduce the critical communication barriers that deaf patients face when seeking urgent medical care. It also contributes to the broader movement toward greater inclusion and accessibility in health care systems.

One of the key outcomes of this study is the development of a clear, structured corpus of terms that can be directly applied not only in the sign language recognition system developed by our group but also in systems created by other research teams. By contributing to the existing body of literature, this work aims to contribute to the development of future systems for medical communication in sign language. The next steps will focus on validating the system through hypothetical medical care scenarios, ensuring that the signs synthesized by the system are accurate and comprehensible for deaf users.

### Limitations

Similar to spoken languages, sign languages are inherently regional and culturally specific. Our work focused on Brazilian Sign Language (Libras), which limits the generalizability of the corpus to sign languages of other countries. While this limitation is intrinsic to any language-based system, future adaptations will require cultural and linguistic tailoring to adapt it to other contexts, thus ensuring broader applicability. However, the primary contribution of this study lies in the methodological approach: a structured, interdisciplinary process for corpus development tailored to health care contexts, rather than in proposing an unabridged compendium of signs.

Second, the number of health care professionals participating in the Delphi process was relatively small. Although their responses were consistent and included a range of clinical experiences, a larger and more diverse sample, including professionals from different regions, more sign language users, and a broader range of professional roles and experiences, could further strengthen the representativeness and validity of the selected terms. Future iterations should aim to broaden participation, possibly involving deaf health care users as well.

Third, the list of terms and questions was based on hypothetical medical emergencies and predefined scenarios. While this approach provided structure and facilitated consensus, it may not fully capture the emotional complexity and spontaneity of real-life interactions in emergency care. Future studies should validate the corpus using real-world consultations or simulations, allowing for refinement based on authentic communication dynamics.

Despite these limitations, we believe that this study proposes a solid foundation for future corpus development efforts. The use of a transparent, interdisciplinary, and consensus-based approach represents a significant methodological advancement in the field of sign language translation systems for health care.

### Potential Scalability and Future Directions

Although this study focused on developing a Brazilian Sign Language (Libras) corpus for emergency care, the methodology and participatory approach used may provide a model for adaptation to other sign languages and cultural contexts. Key elements such as expert consensus on terminology, iterative translation with linguistic validation, and the incorporation of hypothetical clinical scenarios could be applied in different health care settings, allowing for the creation of contextually and culturally appropriate corpora.

Building on this initial effort, future research should focus on validating the proposed corpus in real or simulated health care interactions to assess its communicative adequacy in practice. Further expansions may include broadening the development of corpora for other care contexts, such as primary care or mental health. Additional Delphi rounds involving deaf patients themselves could enrich the corpus with user-centered insights. Moreover, adapting the proposed methodology to other sign languages and national health systems may support the development of culturally relevant corpora worldwide. Finally, the integration of this corpus into translation tools and health professional training programs could enhance accessibility and equity in health care communication.

### Conclusions

This study represents a significant methodological step toward improving communication between health care professionals and deaf individuals in emergency medical settings. Rather than proposing a universal solution, the study presents a structured and participatory approach for developing a corpus of medical terms in Libras, with interdisciplinary validation. The process included the involvement of deaf sign language experts during the translation and linguistic adaptation phase, ensuring that the corpus reflects authentic usage and articulation in Libras.

The findings underscore the complexity of translating medical terminology into sign language and highlight the importance of integrating both health care and linguistic expertise. While this corpus provides a solid foundation, future validation in real-world or simulated scenarios remains essential to assess its practical utility.

## Supplementary material

10.2196/72789 Multimedia Appendix 1 Questions included in the questionnaire.

10.2196/72789 Multimedia Appendix 2 Importance scores assigned by participants with previous emergency services experience to each question proposed for deaf individuals’ health care.

10.2196/72789 Multimedia Appendix 3 Final version of the questions and their respective videos in sign language.

10.2196/72789 Multimedia Appendix 4 Hypothetical medical care situations.

## Abbreviations

Libras: Língua Brasileira de Sinais
UFMG: Universidade Federal de Minas Gerais
WHO: World Health Organization
