# Supplementary material for: On the Design of a Sign Language Corpus of Medical Terms for Automatic Translation Systems: Mixed Methods Approach
Source: JMIR Hum Factors. 2026 Apr 29;13:e72789. doi: 10.2196/72789 (PMC13127854; doi:10.2196/72789)
Supplement: Multimedia Appendix 1 [file humanfactors-v13-e72789-s001.docx]

Questions included in the questionnaire.

| **Professional profile questions** |
| --- |
| 1. Are you: (1) a doctor (2) a nurse |
| 2. Do you work in an emergency service? |
| 3. What is the setting of the emergency service you work in? |
| 4. How long have you worked in an emergency service (years)? |
| 5. Have you ever treated a patient with hearing impairment in an emergency situation? |
| 6. If yes to the question above: how many patients? |
| **Likert scale questions** |
| 7. For each question below, rate it from 0 to 5, from least to most important: |
| a. What are you feeling that made you seek the hospital? |
| b. When did it start? |
| c. How did it start? What were you doing when it started? |
| d. How long has it lasted? |
| e. Do you feel anything else, besides this symptom? |
| f. Is there any factor that makes it worse or better? |
| g. If the complaint is chest pain or shortness of breath: does it get worse when you make a physical effort? Climbing a hill, walking on a flat surface, taking a shower? Or does it appear when you are at rest, without any effort? Has it improved with any medication? |
| h. If the complaint is pain: Where is the pain? What is the pain like? Does it feel like a tightness or weight, or burning, or stabbing, or a shock? |
| i. On a scale of 1 to 10, with 1 being very weak pain and 10 being unbearable pain, what is the intensity of the pain? |
| j. Does it stay in one fixed place, or does it spread to another location? |
| k. Does it get worse when you press? Does it get worse when you change your body position? |
| l. If you complained of fever or chills, do you have sneezing or nasal discharge? |
| m. Do you have a cough? If so, what is the cough like, productive or dry? What color is the phlegm? |
| n. If you don't sneeze or cough, do you feel a burning sensation when urinating? |
| o. If you complained of fever, chills, cough, shortness of breath, or if you said you had sneezing or nasal discharge: have you been vaccinated against COVID-19? If so, how many doses? Have you been vaccinated against the flu? |
| p. Are you being treated for any disease? If so, which one? |
| q. Are you allergic to any medication? If so, which one? |
| r. Did you bring a medical report? |
| s. Did you bring a prescription? If not, are you taking any medication? |
| t. Do you smoke? |
| u. Do you drink alcohol? If so, how much? |
| **Open-ended questions** |
| 8. Do you think that one or more questions should have their writing changed? If so, which ones? |
| 9. Do you have any other suggestions for questions or other general suggestions? Feel free to make any comments you consider important. |
| 10. Do you agree to be contacted, if necessary, to better understand the suggestions? |
| 11. If so, what is your email address? |
