# Supplementary material for: On the Design of a Sign Language Corpus of Medical Terms for Automatic Translation Systems: Mixed Methods Approach
Source: JMIR Hum Factors. 2026 Apr 29;13:e72789. doi: 10.2196/72789 (PMC13127854; doi:10.2196/72789)
Supplement: Multimedia Appendix 2 [file humanfactors-v13-e72789-s002.docx]

| **Multimedia Appendix 2.** Importance scores assigned, by participants with previous emergency services experience, to each question proposed for deaf individuals’ health care* | | | | | | |
| --- | --- | --- | --- | --- | --- | --- |
| Questions | Score 1 (%) | Score 2 (%) | Score 3 (%) | Score 4 (%) | Score 5 (%) | Median  (IQR) |
| 1. What are you feeling that made you seek the hospital? | 0 | 0 | 8.33 | 16.67 | 75 | 5 (4-5) |
| 2. When did it start? | 0 | 8.33 | 33.33 | 8.33 | 50 | 5 (3-5) |
| 3. How did it start? What were you doing when it started? | 0 | 8.33 | 33.33 | 8.33 | 50 | 5 (3-5) |
| 4. How long has it lasted? | 0 | 0 | 25 | 25 | 50 | 5 (3-5) |
| 5. Do you feel anything else, besides this symptom? | 0 | 0 | 25 | 16.67 | 58.33 | 5 (3-5) |
| 6. Is there any factor that makes it worse, or better? | 0 | 8.33 | 25 | 25 | 41.67 | 4 (3-5) |
| 7. If the complaint is chest pain or shortness of breath: does it get worse when you make a physical effort? Climbing a hill, walking on a flat surface, taking a shower? Or does it appear when you are at rest, without any effort? Has it improved with any medication? | 0 | 0 | 16.67 | 16.67 | 66.67 | 5 (4-5) |
| 8. If the complaint is pain: Where is the pain? What is the pain like? Does it feel like a tightness or weight, or burning, or stabbing, or a shock? | 0 | 8.33 | 16.67 | 25 | 50 | 4 (3-5) |
| 9. On a scale of 1 to 10, with 1 being very weak pain and 10 being unbearable pain, what is the intensity of the pain? | 0 | 16.67 | 16.67 | 25 | 41.67 | 4 (3-5) |
| 10. Does it stay in one fixed place, or does it spread to another location? | 0 | 8.33 | 41.67 | 8.33 | 41.67 | 3 (3-5) |
| 11. Does it get worse when you press? Does it get worse when you change your body position? | 0 | 0 | 41.67 | 25 | 33.33 | 4 (3-5) |
| 12. If you complain of fever or chills, do you have sneezing or nasal discharge? | 0 | 8.33 | 16.67 | 33.33 | 41.67 | 4 (3-5) |
| 13. Do you have a cough? If so, what is the cough like, productive or dry? What color is the phlegm? | 0 | 0 | 33.33 | 33.33 | 33.33 | 4 (3-5) |
| 14. If you don't sneeze or cough, do you feel a burning sensation when urinating? | 0 | 16.67 | 33.33 | 25 | 25 | 4 (3-5) |
| 15. If you complained of fever, chills, cough, shortness of breath, or if you said you had sneezing or nasal discharge: have you been vaccinated against COVID-19? If so, how many doses? Have you been vaccinated against the flu? | 0 | 16.67 | 33.33 | 16.67 | 33.33 | 4 (3-5) |
| 16. Are you being treated for any disease? If so, which one? | 0 | 8.33 | 33.33 | 16.67 | 41.67 | 4 (3-5) |
| 17. Are you allergic to any medication? If so, which one? | 0 | 0 | 16.67 | 16.67 | 66.67 | 5 (4-5) |
| 18. Did you bring a medical report? | 8.33 | 8.33 | 33.33 | 25 | 25 | 3 (3-5) |
| 19. Did you bring a prescription? If not, are you taking any medication? | 0 | 8.33 | 33.33 | 16.67 | 41.67 | 4 (3-5) |
| 20. Do you smoke? | 0 | 25 | 25 | 25 | 25 | 3 (2-5) |
| 21. Do you drink alcohol? If so, how much? | 0 | 16.67 | 33.33 | 8.33 | 41.67 | 3 (3-5) |

* A Likert scale ranging from 1 to 5 was used, with the following correspondence: 1 = not important at all; 2 = of little importance; 3 = of average importance; 4 = very important; 5 = absolutely essential.

IQR: interquartile range
