# Supplementary material for: On the Design of a Sign Language Corpus of Medical Terms for Automatic Translation Systems: Mixed Methods Approach
Source: JMIR Hum Factors. 2026 Apr 29;13:e72789. doi: 10.2196/72789 (PMC13127854; doi:10.2196/72789)
Supplement: Multimedia Appendix 3 [file humanfactors-v13-e72789-s003.docx]

Final version of the questions and their respective videos in sign language.

| **Questions** | **Sign language video sentences** |
| --- | --- |
| What are you feeling that made you seek the hospital? | <https://youtu.be/8eP5JNzRIQE> |
| When did it start? | <https://youtu.be/rIehnPdd-dE> |
| How did it start? What were you doing when it started? | <https://youtu.be/yw-DdNgfQ7U> |
| How long has it lasted? | <https://youtu.be/9r8en-Shv04> |
| Do you feel anything else, besides this symptom? | <https://youtu.be/LlOAbH64Ktg> |
| Is there any factor that makes it worse or better? | *Is there any factor that makes it worse?*  <https://youtu.be/1_Sck18hOsM> |
|  | *Is there any factor that makes it   better?*  <https://youtu.be/ZrxZUi22XfM> |
| If the complaint is chest pain or shortness of breath: does it get worse when you make a physical effort? Climbing a hill, walking on a flat surface, or taking a shower? Or does it appear when you are at rest, without any effort? Has it improved with any medication? | *Does the chest pain or shortness of breath get worse when you make a physical effort?* <https://youtu.be/Eq0c_9ClxXI> |
|  | *Does the pain get worse when climbing a hill?*  <https://youtu.be/qiMQInOfJeA> |
|  | *Does the pain get worse when walking on a flat surface?* <https://youtu.be/CO15D9C6xaQ> |
|  | *Does the pain get worse when taking a shower?*  <https://youtu.be/q8O4QpEiSow> |
|  | *Does the pain get worse when you are at rest?*  <https://youtu.be/LTYvqOv9xm0> |
|  | *Has the chest pain or shortness of breath improved with any medication?*  <https://youtu.be/kTIAmn7a1wU> |
| If the complaint is pain: Where is the pain? What is the pain like? Does it feel like a tightness or weight, or burning, or stabbing, or a shock? | *Where is the pain?*  <https://youtu.be/Aq5VJAXpsmg> |
|  | *What is the pain like?*  <https://youtu.be/XpJBuj2t5ng> |
|  | *Does the pain feel like a tightness or weight?*  <https://youtu.be/Z-D2Oghsbng> |
|  | *Does the pain feel like a burning?* <https://youtu.be/rI2b1XppuS8> |
|  | *Does the pain feel like a stabbing?*  <https://youtu.be/ty7hkBBfPpw> |
|  | *Does the pain feel like a shock?*  <https://youtu.be/NqhcTg3VTqI> |
| On a scale of 1 to 10, with 1 being very weak pain and 10 being unbearable pain, what is the intensity of the pain? | <https://youtu.be/IffLSW1e02w> |
| Does it stay in one fixed place, or does it spread to another location? | <https://youtu.be/YhI2Jh5nKHs> |
| Does it get worse when you press? Does it get worse when you change your body position? | *Does the pain get worse when you press?*  <https://youtu.be/ETFQBHN7ojo> |
|  | *Does the pain get worse when you change your body position?*  <https://youtu.be/DPJaKPWGvis> |
| If you complain of fever or chills, do you have sneezing or nasal discharge? | <https://youtu.be/7v4ukhPv0l8> |
| Do you have a cough? If so, what is the cough like, productive or dry? What color is the phlegm? | *Do you have a cough?*  <https://youtu.be/TOpmMc81Goc> |
|  | *What is the cough like?*  <https://youtu.be/Q2oAYIn_474> |
|  | *Is the cough productive or dry?*  <https://youtu.be/30Cu0DsM7IY> |
|  | *What color is the phlegm?*  <https://youtu.be/f2-3HFc-3WY> |
| If you don't sneeze or cough, do you feel a burning sensation when urinating? | <https://youtu.be/0-V1fcKGAPk> |
| If you complained of fever, chills, cough, shortness of breath, or if you said you had sneezing or nasal discharge: have you been vaccinated against COVID-19? If so, how many doses? Have you been vaccinated against the flu? Did you bring your vaccination card? | *Have you been vaccinated against COVID-19?*  <https://youtu.be/7Cd2DARC_vI> |
|  | *How many doses?*  <https://youtu.be/UNRAD8ukm10> |
|  | *Have you been vaccinated against the flu?*  <https://youtu.be/x_3MovtBMqQ> |
|  | *Did you bring your vaccination card?*  <https://youtu.be/vWFcMnWn4jc> |
| Are you being treated for any disease? If so, which one? | <https://youtu.be/XGoyVAHbcW4> |
| Do you have any allergies? Are you allergic to any medication? If so, which one? | *Do you have any allergies?*  <https://youtu.be/slvf2BOpn8k> |
|  | *Are you allergic to any medication?*  <https://youtu.be/3I9FOEJt9Nw> |
|  | *Which medication?*  <https://youtu.be/ntvtjWjtnTA> |
| Did you bring a medical report? | <https://youtu.be/Ee4hsArtBEo> |
| Did you bring a prescription? If not, are you taking any medication? | *Did you bring a prescription?*  <https://youtu.be/FJiF9E5HgFU> |
|  | *Are you taking any medication?*  <https://youtu.be/5TDszm26ESk> |
| Do you smoke? If so, for how long and how many cigarettes per day? | *Do you smoke?*  <https://youtu.be/5yIj2yFlcj8> |
|  | *How long do you smoke?*  <https://youtu.be/Rm_GpVQRbFw> |
|  | *How many cigarettes per day?*  <https://youtu.be/pwetQo308BU> |
| Do you drink alcohol? If so, which drink(s) and how much? | *Do you drink alcohol?*  <https://youtu.be/m1rVOBqiC6o> |
|  | *Which drink?*  <https://youtu.be/KxFdyT71m_E> |
|  | *How much do you drink?*  <https://youtu.be/_D4q2uj_krU> |
