# Supplementary material for: On the Design of a Sign Language Corpus of Medical Terms for Automatic Translation Systems: Mixed Methods Approach
Source: JMIR Hum Factors. 2026 Apr 29;13:e72789. doi: 10.2196/72789 (PMC13127854; doi:10.2196/72789)
Supplement: Multimedia Appendix 4 [file humanfactors-v13-e72789-s004.docx]

Hypothetical medical care situations.

Scenario 1. Heart attack:

Chest pain, like a tightness, spreads to the left arm, neck, and jaw, with an intensity of 10 out of 10. It started 15 minutes ago, after physical activity, and is associated with sweating and palpitations (a pounding sensation in the chest). The pain improves with rest and worsens with physical activity.

Scenario 2. Appendicitis:

Cramping pain in the middle of the abdomen, close to the navel, which develops into a stabbing pain in the right groin area. It started at rest, 30 minutes ago, and is associated with fever, sweating, palpitations (a pounding sensation in the chest) and nausea. The pain worsens when stretching the leg and when pressing the right groin area.

Scenario 3. Nephrotic (Renal) Colic:

Cramping pain in the lower back, which spreads to the groin on the same side, with an intensity of 10 out of 10. It started 10 minutes ago and is associated with nausea, vomiting, and bloody urine (dark urine). There is no improvement or worsening factor.

Scenario 4. Anaphylactic Reaction:

Difficulty breathing, swelling, and skin spots, which began 20 minutes ago, after consuming peanuts and seafood. There is no improvement or worsening factor.

Scenario 5. Migraine:

Pulsing (throbbing) head pain on one side of the head, intensity 8 out of 10, which was preceded by spots in the vision, nausea, and tingling. It started 30 minutes ago at rest and worsens with physical activity and when being in noisy and brightly lit places. The pain improves with complete rest.

Scenario 6. Pneumonia:

High fever, started 3 days ago, preceded by sneezing and runny nose (nasal secretion) that did not improve with treatment. It is associated with difficulty breathing and a productive cough, with phlegm that was initially clear and gradually became yellow-green.

Scenario 7. Cystitis:

Painful, burning sensation when urinating, which started 2 days ago, after resuming sexual activity. Associated with an increased need to go to the bathroom and pain when pressing on the lower abdomen (bladder area). It improves with increased water intake.

Scenario 8. Decompensated Heart Failure:

Breathing difficulty, which started 4 days ago, when walking on a flat surface and has been getting worse over time, now occurring even at rest. It is associated with swelling in the legs and worsens when lying down.

Scenario 9. Exacerbated Chronic Obstructive Pulmonary Disease (COPD):

Breathing difficulty worsened 2 days ago. It is associated with worsening cough and increased expectoration (sputum), with yellow-green phlegm. History of smoking cigarettes for over 30 years, with approximately 2 packs per day.

Scenario 10. Erysipelas:

Well-defined reddish area on the leg, which appeared 3 days ago, after a local injury. It is associated with pain when pressing the region, local swelling and heat. There is no sensation of a hardened calf.

Scenario 11. Infectious Diarrhea:

Liquid, clear and explosive diarrhea started 2 days ago, after consuming spoiled mayonnaise. Presence of blood, mucus, pus, and food debris in the stool. It is associated with fever, nausea, vomiting, increased thirst, and cramping pain in the middle of the abdomen.

Scenario 12. Cholecystitis:

Cramping pain in the upper right side of the abdomen, started 4 days ago, with an intensity 7 out of 10. It is associated with nausea, vomiting, fever, loss of appetite, and bright diarrhea. It gets worse with the consumption of fatty foods and when pressing the abdomen area.

Scenario 13. Deep Vein Thrombosis:

Pain in the leg started 10 minutes ago, intensity of 8 out of 10. It is associated with local swelling and heat. There is a sensation of a hardened calf.

Scenario 14. Asthma Attack:

Difficulty breathing, started 25 minutes ago, after entering a dusty environment with people smoking. It is associated with wheezing in the chest. History of allergic rhinitis and use of “inhalers”.
